# Seroprevalence for dengue virus in a hyperendemic area and associated socioeconomic and demographic factors using a cross-sectional design and a geostatistical approach, state of São Paulo, Brazil

**DOI:** 10.1186/s12879-019-4074-4

**Published:** 2019-05-20

**Authors:** Francisco Chiaravalloti-Neto, Rafael Alves da Silva, Nathalia Zini, Gislaine Celestino Dutra da Silva, Natal Santos da Silva, Maisa Carla Pereira Parra, Margareth Regina Dibo, Cassia Fernanda Estofolete, Eliane Aparecida Fávaro, Karina Rocha Dutra, Manlio Tasso Oliveira Mota, Georgia Freitas Guimarães, Ana Carolina Bernardes Terzian, Marta Blangiardo, Mauricio Lacerda Nogueira

**Affiliations:** 1 Departamento de Epidemiologia, Faculdade de Saúde Pública, Universidade de São Paulo (USP), Avenida Doutor Arnaldo 715, São Paulo, SP 01246-904 Brazil; 2 Laboratório de Pesquisas em Virologia, Departamento de Doenças Dermatológicas Infecciosas e Parasitárias, Faculdade de Medicina de São José do Rio Preto (FAMERP), Avenida Brigadeiro Faria Lima, 5416, São José do Rio Preto, SP 15090-000 Brazil; 3 Laboratório de Modelagens Matemática e Estatística em Medicina, Faculdade de Medicina, União das Faculdades dos Grandes Lagos, Rua Doutor Eduardo Nielsen 960, São José do Rio Preto, SP 15030-070 Brazil; 4 Laboratório de Entomologia, Superintendência de Controle de Endemias, Rua Cardeal Arcoverde 2878, São Paulo, SP 05408-003 Brazil; 5 MRC-PHE Centre for Environment and Health, Department of Epidemiology and Biostatistics, Imperial College, St. Mary’s Campus, Norfolk Place, London, W2 1PG UK

**Keywords:** Dengue, Seroprevalence, Cross-sectional study, Geostatiscal analysis, Brazil

## Abstract

**Background:**

São José do Rio Preto is one of the cities of the state of São Paulo, Brazil, that is hyperendemic for dengue, with the presence of the four dengue serotypes.

Objectives: to calculate dengue seroprevalence in a neighbourhood of São José do Rio Preto and identify if socioeconomic and demographic covariates are associated with dengue seropositivity.

**Methods:**

A cohort study to evaluate dengue seroprevalence and incidence and associated factors on people aged 10 years or older, was assembled in Vila Toninho neighbourhood, São José do Rio Preto. The participant enrolment occurred from October 2015 to March 2016 (the first wave of the cohort study), when blood samples were collected for serological test (ELISA IgG anti-DENV) and questionnaires were administrated on socio-demographic variables. We evaluated the data collected in this first wave using a cross-sectional design. We considered seropositive the participants that were positive in the serological test (seronegative otherwise). We modelled the seroprevalence with a logistic regression in a geostatistical approach. The Bayesian inference was made using integrated nested Laplace approximations (INLA) coupled with the Stochastic Partial Differential Equation method (SPDE).

**Results:**

We found 986 seropositive individuals for DENV in 1322 individuals surveyed in the study area in the first wave of the cohort study, corresponding to a seroprevalence of 74.6% (95%CI: 72.2–76.9). Between the population that said never had dengue fever, 68.4% (566/828) were dengue seropositive. Older people, non-white and living in a house (instead of in an apartment), were positively associated with dengue seropositivity. We adjusted for the other socioeconomic and demographic covariates, and accounted for residual spatial dependence between observations, which was found to present up to 800 m.

**Conclusions:**

Only one in four people aged 10 years or older did not have contact with any of the serotypes of dengue virus in Vila Toninho neighbourhood in São José do Rio Preto. Age, race and type of house were associated with the occurrence of the disease. The use of INLA in a geostatistical approach in a Bayesian context allowed us to take into account the spatial dependence between the observations and identify the associated covariates to dengue seroprevalence.

**Electronic supplementary material:**

The online version of this article (10.1186/s12879-019-4074-4) contains supplementary material, which is available to authorized users.

## Background

Dengue (DENV) is an arbovirus transmitted by hematophagous mosquitoes of *Aedes* genus, mainly *Aedes aegypti,* and presents four serotypes: DENV-1to 4. The disease caused by DENV is considered an important world public health problem, mainly in tropical and subtropical regions, both in terms of morbidity and mortality and is endemic in more than 125 countries. Estimates point out a world incidence around 80 million cases for 2015, amount 2.5 times the incidence estimated for 2005, and 10,000 deaths per year [1–6]. Brazil presented in recent years a sharp increase in dengue incidence and number of deaths, a picture related to the multiple viral circulation, and accounts for around 70% of the dengue cases in Americas [7].

The development of studies to identify the determinants of DENV infection is of fundamental importance for the definition of preventive measures and for the structuring of adequate surveillance systems and effective control programs [8]. However, most of them are based on information obtained from the reporting systems which are based on cases confirmed by laboratory and clinical-epidemiological criteria [9, 10]. This causes important limitations for these type of studies, since they are based on symptomatic cases, are subject to underreporting and do not take into account that a large proportion of dengue infections are asymptomatic [9, 11–14].

Instead, seroepidemiological studies are not subjected to the limitations of the investigations based on incidence data reporting systems and provide a more realistic picture of the disease burden in the population. In addition, they are considered useful for evaluating the effectiveness of control measures, including the establishment of vaccination strategies and evaluation of their effectiveness [9, 14, 15]. Recently a dengue vaccine has been licensed in some countries in Latin America and Asia with the recommendation to be used only in endemic and high seroprevalence regions for dengue [16]. In countries where this strategy is being or will be considered, the development of seroepidemiological studies are or will be fundamental for the definition of the regions and population groups to be vaccinated.

Despite their importance, Imai et al. [9] report the relative rarity of seroepidemiological studies compared to the volume of studies based on data provided by surveillance systems. A fact that can be attributed to the limited resources for their development in countries where dengue is endemic [17]. The picture is not different in Brazil and in the state of São Paulo. According to the literature review we conducted in scientific journals, we identified in Brazil, eight seroepidemiological studies conducted in the 1990s [11, 18–24], seven in the 2000s [25–31] and only three in the 2010s [32–34]. From these study, only three were developed in the state of São Paulo, all of them in the 1990s [21–23].

Considering the rarity of this type of study in Brazil, especially in the 2010s, and on the other hand, its epidemiological importance, we highlight the present seroprevalence study carried out in the state of São Paulo. It was developed, as part of a dengue prospective cohort study, to calculate the seroprevalence of dengue in a neighbourhood of the municipality of São José do Rio Preto and to identify if socioeconomic and demographic covariates are associated with the dengue seropositivy.

## Methods

### Study area, type and period

**Fig. 1:**
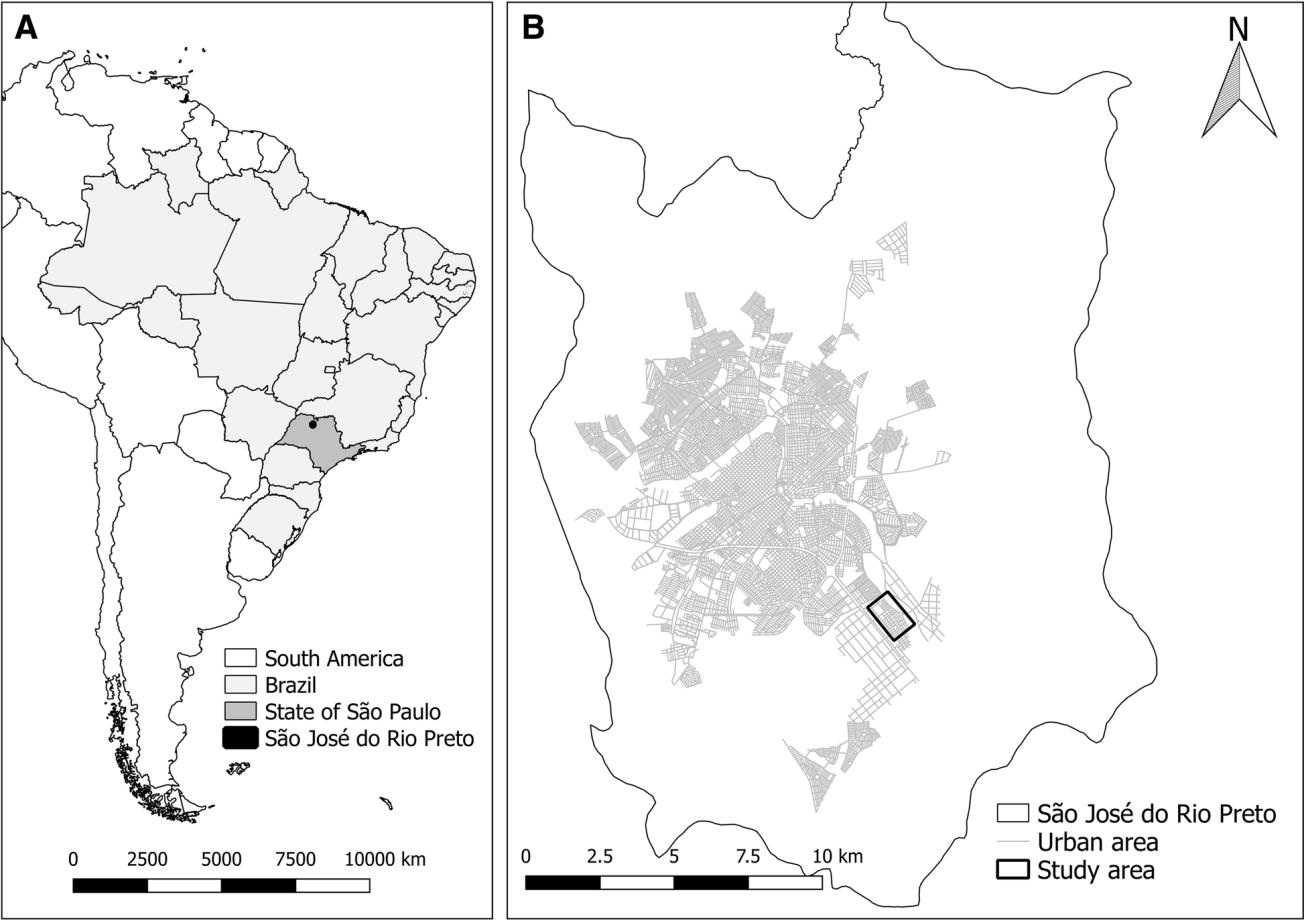
**a**: Municipality of São José do Rio Preto, state of São Paulo, Brazil, South America; **b**: Vila Toninho neighborhood (study area) in the municipality of São José do Rio Preto

The study was developed in the municipality of São José do Rio Preto, state of São Paulo, Brazil (Fig. 1). The city is located in the country’s southwestern region in latitude 20°49′11 “S and longitude 49°22′46 “W, has a population of around 450,000 inhabitants, tropical climate with temperature and average annual rainfall of 25 °C and 1410 mm, respectively, and altitude of 475 m asl. The HDI (Human Development Index) of São José do Rio Preto is 0.797, one of the highest in Brazil (52th position among the 5565 Brazilian municipalities) (www.ibge.gov.br).

We selected, as the study area, an area belonging to Vila Toninho neighborhood located in the southeastern part of the urban part of the city (Fig. 1b), with 5575 inhabitants, 4809 with 10 years or older (2322 male and 2487 female), 1937 residences and 4806 inhabitants per km^2^. Vila Toninho is located in the outskirts of São José do Rio Preto and has socioeconomic indicators worse than the city averages: 87.9% of people with five or more years are literate, average income of head of household of 1.9 Brazilian minimum salaries (MS) and 15.3% of the household with five or more residents (against, respectively, 95.9%, 5.7 MS and 11.5% for the entire municipality) (www.ibge.gov.br).

The reinfestation by *Ae. aegypti* in São José do Rio Preto was detected in 1985 (Chiaravalloti-Neto 1997), the first autochthonous cases by DENV-1 occurred in 1990 and the first dengue epidemic occurred on 1994–1995. DENV-2, DENV-3 and DENV-4 were detected, respectively in 1998, 2005 and 2011, causing new epidemics, so that the city is considered hyperendemic for dengue fever [35, 36]. In the 2010s, three major dengue epidemics occurred in the city: 25 thousand cases in 2010, 19 thousand in 2013 e 22 thousand in 2015. Even though these epidemics have reached all over the city, one of their characteristics has been present higher incidences in the most peripheral and the poorest area of São José do Rio Preto, including Vila Toninho neighbohood. Taking this in account, we chose this neighbourhood as study area because it well represents the most susceptible regions of the city for the occurrence of dengue [35, 37, 38].

A cohort to investigate dengue seroprevalence and seroincidence and associated factors was assembled in the study area and the participants were enrolled between October 2015 and March 2016 (the cohort first wave). Besides the first wave, the cohort includes more three waves to measure the seroincidences (one measure per year). The present study is a cross-sectional analysis of the data collected during the cohort first wave.

### Study population

The eligibility criteria to participate of the cohort were to be 10 years or older and to be a resident of Vila Toninho neighbourhood. To obtain the sample size necessary for the development of the study, we estimated that 380 seronegative individuals would be necessary to measure a seroincidence of 10%, with an error margin of 3% and a significance level of 5%, by the fourth and final wave of the cohort. We established the initial sample initial size around 1400 individuals considering that we would also have seroincidences of 10% and annual losses of 10% in the first, second and third waves of the cohort and a seroprevalence of 50% in the first wave.

### Data collection

We visited all households in the study area and, in those that were not closed during our visits, all residents aged 10 years or older were invited to participate in the cohort. After agreement and signature of the Informed Consent Form, the study participant answered a structured questionnaire about their sociodemographic characteristics and past dengue occurrence. The questionnaire also included questions on the knowledge of the disease, vector and control measures, issues to be addressed in future publications. Two blood tubes were then collected from the participant, one for the collection of whole blood (with K2 EDTA 7.2 mg anticoagulant (BD Vacutainer®)) and another to obtain serum (SST™ BD Vacutainer® tubes). The tubes were sent under refrigeration to the Laboratory of Virology of School of Medicine of São José do Rio Preto (FAMERP), centrifuged at a rate of 2000 g for 10 min and stored in a freezer at − 80 °C until. The period we made these blood collections was before the Zika virus circulation in the city.

Serum exams of the study participants were performed using the anti-DENV IgG ELISA method, using the commercial kit Human Anti-Dengue vírus IgG ELISA kit (Abcam®, Cambridge, United Kingdom) and following the manufacturer’s instructions. The optical density (OD) reading was performed at an absorbance of 450 nm with the equipment Spectramax Plus ELISA reader (Molecular Devices©) and the calculations were made according to the kit instructions.

The residential addresses of the individuals enrolled in the study were geocoded using a georeferenced street map that was available by the São José do Rio Preto City Council. Then, for each enrolled individual we obtained their plain Cartesian coordinates in meters in the UTM coordinate system, 22S zone, Datum SIRGAS 2000. We attributed a code for each participant and its collected data (questionnaire, serological test result and Cartesian coordinates) were typed in a Microsoft Excel spreadsheet.

### Variables

The study dependent variable was the seropositivity for dengue (IgG). We considered as seropositive (IgG pos), a participant who presented a positive test for dengue in the anti-DENV IgG ELISA serological test and seronegative (IgG neg), otherwise. We considered the following categorical covariates as predictors: sex (male or female), race (white or non-white), marital status (not married or married), occupation (inside or outside); schooling (<=7 or > 7 years), house type (apartment or house), home ownership (owner or non-owner), hours at home (<=12 h or > 12 h per day) and number of residents (<=4 or > 4 residents in the house). We also considered the standardized continuous covariates age and income as predictors. The standardization consisted in subtracting from the values of these covariates the respective means, followed by the division by the respective standard deviations.

### Statistical analysis

We used dot plot charts to identify possible outliers, calculated the percentage of missing data for each variable and evaluated the collinearity between predictors using the variance inflation factor (VIF), using a threshold equal to 3 [39]. As we had missing data in several covariates, we used the multivariate imputation by chained equation methods for the imputation, under the assumption that the missingness occurred at random [40]. For the imputation we used R Statistical software [41] and the mice package [40].

To model the dengue seropositivity (IgG) we specified a Bernoulli probability distribution (eqs. 1 and 2) in a Bayesian context:

1 $$ {IgG}_i\sim Bern\left({\pi}_i\right) $$

2 $$ logit\left({\pi}_i\right)=\alpha +\sum \limits_{p=1}^p{\beta}_p{X}_{pi}+W\left({\boldsymbol{s}}_i\right) $$

where i = 1,...,N represents the ID of a particular individual; π_i_ is the probability of a individual to be dengue seropositive; α is the intercept; **β** is the vector of P regression parameters for the predictors; **X** is the matrix of predictors. Finally, **s**_i_ are the Cartesian coordinates of the individual residential location and W(s_i_) is a realization of a latent stationary Gaussian field (GF) that models the spatial dependence between the address locations of the participants (Cartesian coordinates):

$$ \mathbf{W}\sim \mathrm{MVN}\left(0,\boldsymbol{\Sigma} \right). $$

We modelled the spatially structured covariance matrix **Σ** by a Matérn function [42] (Cressie 1993), considering the Euclidean distance between the individual locations. The GF was represented by a Gaussian Markov random field (GMRF) and we applied the Integrated Nested Laplace Approximations (INLA) approach [43] and coupled with the stochastic partial differential equation (SPDE) for Bayesian inference using the R statistical software [41] and R-INLA package (www.r-inla.org).

We ran the following models: (i) including only the intercept (*intercept non-spatial model*); (ii) including the intercept and the spatial component (*intercept spatial model*); (iii) including the intercept and covariates for the non-imputed dataset (*complete covariate non-spatial model*); (iv) including the intercept, covariates and the spatial component for the non-imputed dataset (*complete covariate spatial model*); (v) including the intercept and covariates for the imputed dataset (*imputed covariate non-spatial model*); (vi) including the intercept, covariates and the spatial component for the imputed dataset (*imputed covariate spatial model*). The process to impute the missing data [40] is the following:

1. We use the *mice* package to regress each variable (with missing) against all the others and obtain five imputed datasets. This was based on Rubin [44], who concluded that *m* equal to five would be sufficient for multivariate imputation.
2. For each imputed dataset we ran model (v) and (vi) described above and obtained the regression coefficients (betas) and their respective standard errors for each one of the covariates.
3. We use Rubin’s rules [44] for combining the estimates into the final ones. This process is explained, in more details, in the Additional file 1.

We ran the six models and used the Deviance Information Criteria (DIC) [45] to compare their goodness of fit. Based on this, in the results we focused on the *imputed covariate spatial model* (model vi) which was characterised by the lowest DIC (corresponding to the best fit); the other models are presented as Additional Materials. After, running model vi without considering interactive effects, we investigated these effects considering all possible interactions among the covariates that were associated with a positive serological result for dengue.

We present the posterior means of the fixed effects and 95% credible intervals (CI) in the logit scale (betas), for models (v) and (vi), and in natural scale (odds ratio (OR)), for model (vi). As this is the first study of dengue seroprevalence in São José do Rio Preto and we did not have prior knowledge about the relationship between the seroprevalence and the covariates, we assumed non-informative priors for the fixed effects in all models and penalized complexity (PC) priors (weak informative priors) for the spatial random effects in the spatial models [46]. The codes for running models (v) and (vi) are presented in Additional file 2.

## Results

**Fig. 2:**
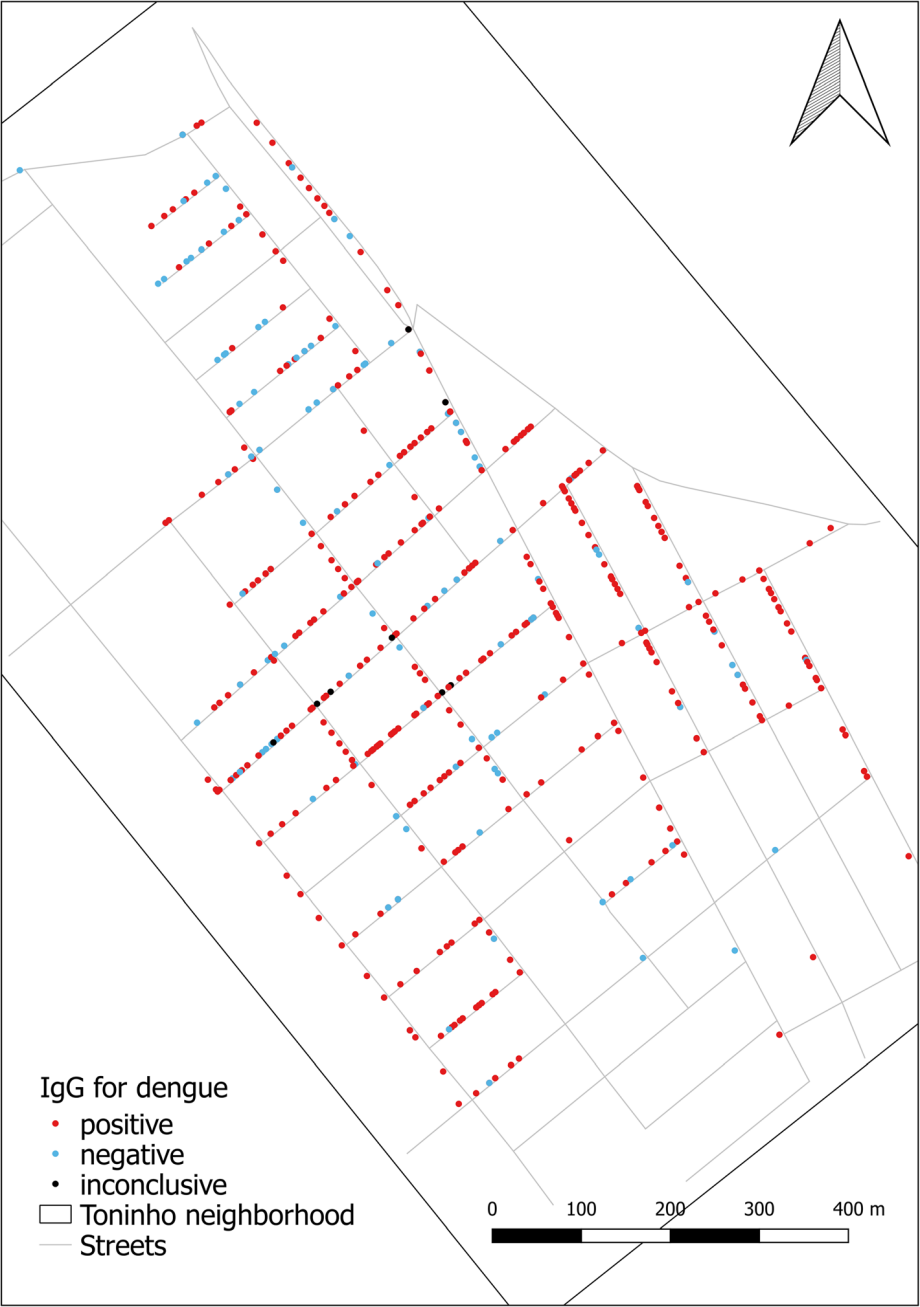
Vila Toninho neighborhood and the distribution of seropositive, seronegative and inconclusive dengue cases, São José do Rio Preto, State of São Paulo, Brazil, 2015–2016

When we concluded the data collection for the first wave of the cohort study, we obtained blood samples for 1347 individual; in Fig. 2 we show the distribution of the IgG test results for dengue across the cohort. Out of 1347 individuals, 25 had inconclusive IgG results for dengue. Thus, we considered in this analysis, 1322 individuals with conclusive IgG results for dengue and geocoded address locations. 986 had positive IgG for dengue, resulting in a seropositivity for dengue of 74.6% (95% CI: 72.2–76.9).

Table 1 presents the distribution of dengue seropositivity across the 1322 individuals (with a conclusive result) split by the covariates included in the statistical model; some of these presented missing data (NA), and in particular income and race had, respectively, 15.3 and 8.1% of NA answers, while the other remaining covariates presented a proportion of missing data lower than 4.0%. We did not identify outliers and collinearity between the covariates. The VIF value considering all covariates was 1.8 (below the chosen threshold). The age and income are presented as categorical in Table 1 and income was categorized in MS.

**Table 1:** Dengue IgG seropositivy by covariates, considering the missing data (NA), Vila Toninho neighborhood, São José do Rio Preto, state of São Paulo, Br, 2015–2016

Covariate	Category	IgG neg		IgG pos		Total (1322; 100%)	
		n	%^a^	n	%^a^	n	%^b^
Sex	Male	130	25.0	391	75.0	521	39.4
	Female	206	25.7	595	74.3	801	60.6
Race	White	197	29.0	482	71.0	679	51.4
	Black and mixed	105	21.6	381	78.4	486	36.8
	Others	11	22.4	38	75.6	49	3.7
	NA	23	21.3	85	78.7	108	8.1
Age (years)	10 to 19	72	36.9	123	63.1	195	14.7
	20 to 39	120	26.3	337	73.7	457	34.6
	40 to 59	112	25.3	331	74.7	443	33.5
	60 to 91	32	14.1	195	85.9	227	17.2
Marital status	Not married	149	26.3	417	73.7	566	42.8
	Married	185	24.9	561	75.2	746	56.4
	NA	2	20.0	8	80.0	10	0.8
Occupation	Inside	68	19.7	277	80.3	345	26.1
	Outside	252	27.0	680	73.0	932	70.5
	NA	16	35.6	29	64.4	45	3.4
Income (minimum salaries – MS)	0 to 2 MS	21	15.6	114	84.4	135	10.2
	2 to 4 MS	221	25.1	660	74.9	881	66.7
	4 MS or more	36	35.0	67	65.0	103	7.8
	NA	58	28.6	145	71.4	203	15.3
Schooling	7 years or less	137	23.0	459	77.0	596	45.1
	8 to 11 years	144	27.8	437	75.2	581	43.9
	More than 12 years	43	45.3	52	54.7	95	7.2
	NA	12	24.0	38	76.0	50	3.8
House type	Apartment	75	34.6	142	65.4	217	16.4
	House	261	23.6	844	76.4	1105	83.6
Home owership	Owner	222	25.6	646	74.4	868	65.7
	Non-owner	109	24.7	332	75.3	441	33.3
	NA	5	38.5	8	61.5	13	1.0
Hours at home (per day)	12 h or less	174	27.6	457	72.4	631	47.7
	More than 12 h	155	23.3	510	76.7	665	50.3
	NA	7	26.9	19	73.1	26	2.0
Number of residents	4 or less	268	26.1	759	73.9	1027	77.7
	More than 4	67	23.2	222	76.8	289	21.9
	NA	1	16.7	5	83.3	6	0.4

^a^row percentages; ^b^ colum percentages

Table 1 also presents the distribution of cohort sample, among which we highlight that it was composed mainly by people of female sex (60.6%), aged between 20 and 59 years old (68.1%), whites (51.4%), married (56.4%), with income less than 4 MS (76.9%), living in houses (83.6%) and only 7.2% with more than 12 years of schooling (upper secondary level of education). Our study also showed that 62.6% (828/1322) of our study population said that never had dengue fever, but among these, 68.4% (566/828) were dengue seropositive.

Our final model, with intercept, covariates and the spatial component for the imputed datasets, is presented in Table 2. Age, race and house type were positively associated with a positive serological result for dengue, after adjustment for socioeconomic and demographic covariates considered in our study and for the spatial dependence between the observations. The combination of the separate estimates that produced these results is presented in Additional file 1. Table 2 also presents the crude OR for the imputed covariate spatial model. The main difference between the crude and adjusted OR is the lack of importance of the covariate Occupation in the final model.

**Table 2:** Posterior mean fixed effects and 95% credible intervals (CI), presented as crude and adjusted odds ratios (OR), of the final model (intercept, covariates and the spatial component for the imputed datasets) (baseline category = 1), Vila Toninho neighbourhood, São José do Rio Preto, state of São Paulo, Brazil, 2015–2016

Covariate	Categories	Posterior mean fixed effects					
		Crude OR			Adjusted OR		
		OR	95%CI		OR	95%CI	
			Lower	Upper		Lower	Upper
Intercept					1.55	0.69	3.46
Sex	Male	1			1		
	Female	0.99	0.76	1.29	0.96	0.73	1.27
Race	White	1			1		
	Non-white	1.36	1.04	1.78	1.42	1.08	1.89
Marital status	Not married	1			1		
	Married	1.16	0.89	1.51	0.93	0.70	1.25
Age (standardized)		1.39	1.21	1.60	1.43	1.21	1.70
Schooling	7 years or less	1			1		
	More than 7 years	0.80	0.61	1.03	1.01	0.76	1.35
Occupation	Internal area	1			1		
	External area	0.70	0.51	0.96	0.91	0.63	1.31
Income (standardized)		0.89	0.78	1.01	0.89	0.78	1.02
House type	Apartment	1			1		
	House	2.04	1.10	3.78	2.02	1.10	3.73
Home ownership	Owner	1			1		
	Non-owner	0.91	0.69	1.21	0.97	0.72	1.31
Hours at home (per day)	12 h or less	1			1		
	More than 12 h	1.17	0.91	1.51	0.95	0.70	1.27
Number of residents	4 or less	1			1		
	More than 4	1.13	0.82	1.55	1.33	0.95	1.86

The investigation of interactive effects in our final model showed that the best fitted model was the one without any interactions (lowest DIC). Also, all the CI 95% for the OR of the interaction terms tested included the unit. We present the DIC values for all fitted models in Additional file 3, where is also possible to see that the models with spatial component presented lower DIC than those without this component and showed better goodness of fit.

The spatial correlation of the models we run for the five imputed databases showed that the maximum distances to where the spatial dependence was present ranged from 848.1 to 919.9 m. The median was 866.1 m and corresponded to the first imputed database; then we show its spatial structure. We obtained a range of around 1000 m for the intercept spatial model (i.e., the spatial model without the covariates), showing that only part of the spatial dependence present in the data was explained by the considered covariates.

**Fig. 3:**
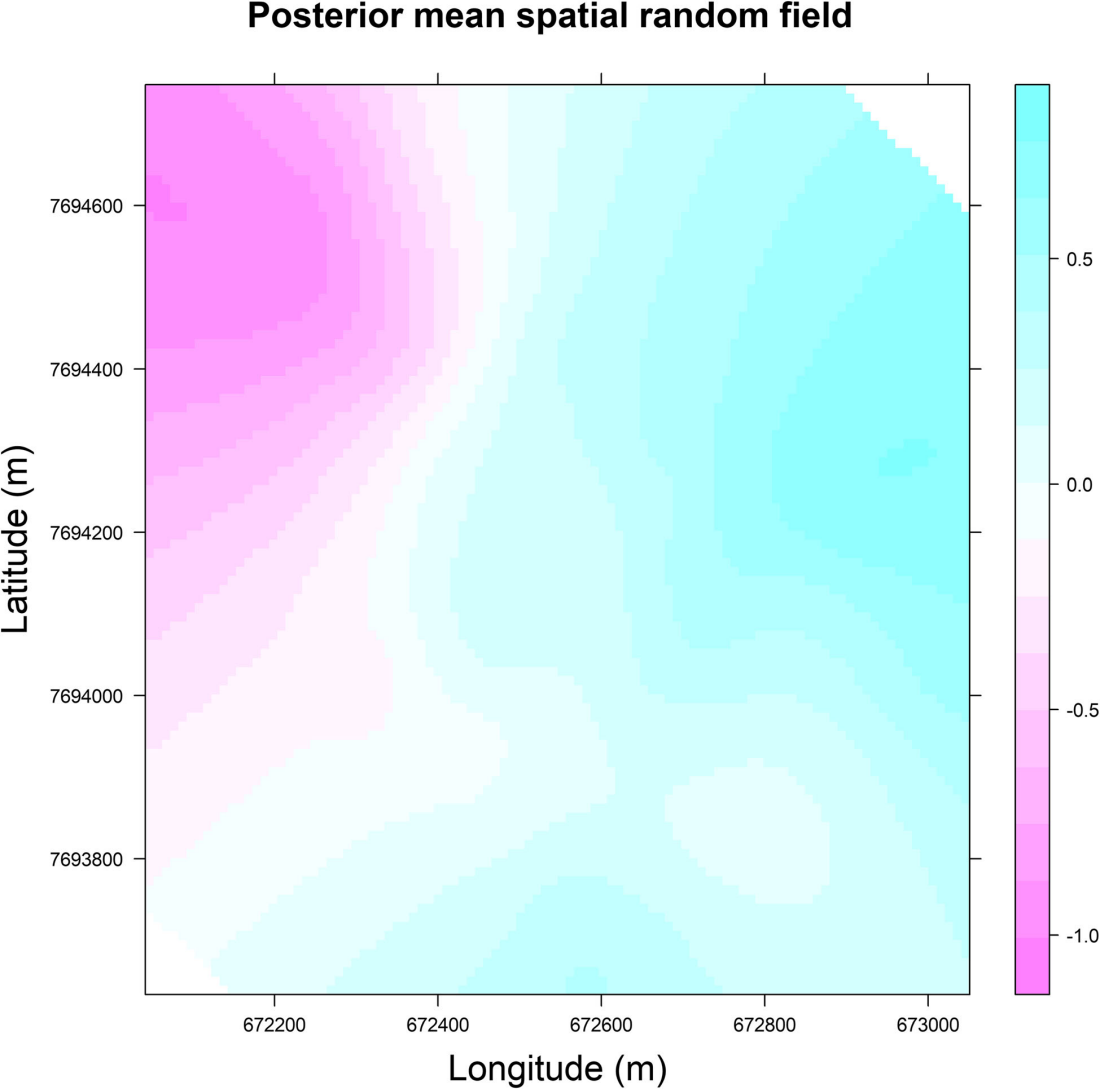
Posterior mean of the spatial random field of the final model (intercept, covariates and the spatial component for the imputed datasets), considering the first imputed database, Vila Toninho neighbourhood, São José do Rio Preto, state of São Paulo, Brazil, 2015–2016

**Fig. 4:**
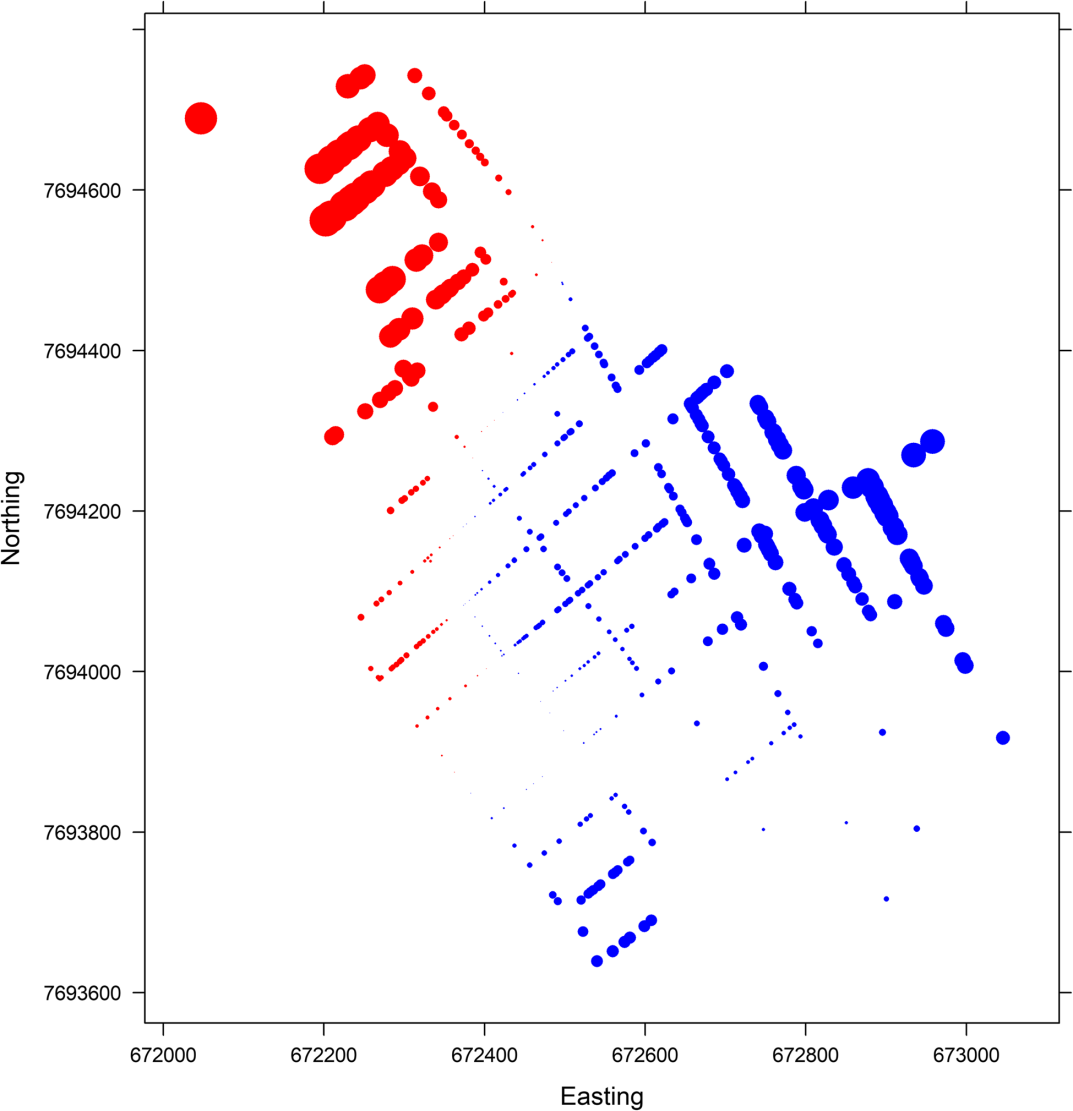
Values of the spatial random field related to the positive and negative dengue cases of the final model (intercept, covariates and the spatial component for the imputed datasets), considering the first imputed database; red colour represents negative values and blue, positive values; Vila Toninho neighbourhood, São José do Rio Preto, state of São Paulo, Brazil, 2015–2016

Figures 3 and 4 present, for the model we run for the first imputed database, respectively, the posterior mean of the spatial random field in the study area and the posterior mean of the spatial random field for the location of the positive and negative dengue cases. These two figures show that the distribution of the spatial random field is not uniform and needs to be considered. In the areas localized in the north-western and western parts of Vila Toninho neighbourhood, the spatial random effect, adjusted for the socioeconomic and demographic covariates, is negative which points towards a reduced probability of occurrence for dengue; in the other areas of the Vila Toninho neighbourhood (excluding that with spatial random effect equal to zero), the spatial random effect, adjust for the same covariates, is positive, meaning that the probability of dengue seropositivity is higher in those areas.

Additional file 4 presents the results of the complete covariate spatial model compared with those of the final model. As the first model was run considering only the individuals with complete data, the sample size diminished to 958, with a loss of 27.5%. The fixed effects of our final model presented narrower 95%CI than the complete spatial model. For this model the house type had a CI95% not including 0, while there was no evidence of an effect of age and race (95%CI including 0). Additional file 5 compares the results of the final model with the imputed covariate non-spatial model and the mean difference between them is that Income lost their importance when the spatial component was considered.

## Discussion

The high seroprevalence that we founded in our study reveals a large burden of disease of DENV in São José do Rio Preto. The seroprevalence found in people aged 10–19 years, slightly lower than the seroprevalences for the other age groups, also reveals high rates of transmission in the recent past. These results can be seen as a consequence of events of the last five and four decades, respectively, in Brazil and the state of São Paulo.

The process of reinfestation of the country by *Ae. aegypti*, after its eradication, began in the 1970s [47] and currently the vector is present in all its States. The first dengue epidemic, post-eradication, occurred in the state of Roraima in 1981 and 1982, with circulation of DENV-1 and DENV-4 [48], followed by the introduction of DENV-1 in Rio de Janeiro in 1986 and its dispersion across several regions of the country [47, 49].

DENV-2 and DENV-3 were also introduced in Rio de Janeiro, respectively in 1990 and 2000, and also dispersed throughout Brazil [47, 50, 51]. Finally, DENV-4 was detected in Manaus, state of Amazon, Northern region of the country in 2005 [52] and, like the other serotypes, dispersed throughout the territory. Simultaneous circulation of the four serotypes generated hyperendemecity and increasing incidences, with a peak in 2015 and occurrence of over 1.6 million cases, and an increase in the number of dengue deaths [53].

*Ae. aegypti* reinfestation in the state of São Paulo was detected in 1985 [54] and the first dengue epidemic occurred in 1990 and 1991, with the circulation of DENV-1 [21]. DENV-2, 3 and 4 were introduced respectively in 1998, 2002 [55] and 2011 [36]. Despite the infestation by the vector in Sao Paulo have been detected only in the 1980s and the first epidemic of dengue have occurred in the early 1990s, the state has had high incidences of dengue and deaths. Among the 1.6 million cases and 1.7 thousand deaths from dengue in Brazil in 2015, around 750 thousand cases and 660 deaths were registered in São Paulo [53].

The comparison of the seroprevalence found in São José do Rio Preto cannot be made with other regions of the State, since our bibliographic survey detected only three studies, all of them developed in the 1990s, with an epidemiological situation distinct from the current situation of the state of São Paulo. The first and second studies were developed after dengue epidemics in Ribeirão Preto (in 1992) and Santa Barbara D’Oeste (in 1995) and obtained seroprevalences, respectively, of 5.4% [21] and 0.6% [22], respectively. The third one was developed in Campinas (in 1998) and obtained a seroprevalence of 14.8% [23].

On the other hand, the seroprevalence found in São José do Rio Preto is in line with the results of two studies carried out in the 2000s and one in the decade of 2010 in Brazilian capitals: between 74.3 and 91.1% in three areas of Recife, state of Pernambuco, between 2005 and 2006 in a population older than 5 years [30]; between 56.1 and 77.4% in three different districts of Rio de Janeiro, state of Rio de Janeiro, in 2007 in a population aged between 1 and 79 years [29]; and 75.7% in Salvador, state of Bahia, between 2015 and 2016 in a population between 0 and 84 years old [34].

The seroprevalence found in São José do Rio Preto also is in line with the values found in studies developed in several Latin American [13, 17, 56–59] and Southeast Asian countries [15, 60].

The similarity between these results, both internal to Brazil as with Latin America and Southeast Asian countries, is related to similar epidemiological realities experienced by the localities considered, that is, a hyperendemic condition with simultaneous circulation of three or four DENV serotypes.

In addition to the high seroprevalence, the high proportion of people with no history of dengue but dengue seropositive in our study also is in line with other studies [11–13]. This confirms that seroepidemiological studies have advantages over those based on surveillance systems, especially because they detect asymptomatic infections [9, 14].

The positive association between age and a positive result for dengue confirms the results of several other studies [11, 15, 26, 29, 30, 57, 58, 61, 62]. The increase in seroprevalence with age is related to a longer period of exposure to the circulating DENV serotypes in endemic areas, that is, older people have had more time to be exposed than younger people [13, 30, 57].

We did not detect an association between income, schooling and number of residents with seropositivity for dengue, variables that can be seen as representative of socioeconomic status. However, seroepidemiological studies [11, 26, 30, 58] and incidence studies based on notification systems [63, 64] have identified a positive association between worse conditions. A hypothesis for the lack of association would be the low variability of these three variables in the study area: around 80% of the participants are characterised by income less than four minimum wages, around 80% of the cohort live in households with four or fewer people and a small proportion of people have reached a higher education.

Among the seroprevalence studies that we found in the scientific literature, none identified an association between race and seropositivity for dengue, and only one showed increased seroconversion in Afro-Colombians compared to whites [13]. Given the homogeneity of the variables representative of the socioeconomic level in the study area and that the non-whites were almost exclusively black and mixed individuals in our study, the fact they showed increased odds for seropositivity for dengue may be a reflection of the worse socioeconomic status of these people in Brazil. A study carried out in Brazil showed that black and mixed-race people presented a higher odds of a negative self-rated health than whites even after controlling for socioeconomic and demographic covariates [65].

Several studies have also found no association between sex and seroprevalence for dengue [13, 15, 26, 30, 59]. Only Amaya-Larios et al. [57] identified an increase in seroprevalence in men. However, they considered this an intriguing result and related it to cultural determinants of the communities, including the specific pattern of human movement of the sites surveyed.

The association between living arrangements (i.e. living in flats has lower risk for dengue) has been detected both in seroepidemiological studies [30, 31] and in incidence studies based on monitoring data [38]. The explanatory factors of this association would be related to less possibility of contact with the vector, since living in apartments means further away from *Ae. aegypti* breeding sites [30]. This affirmation is supported by a study developed in the same city of the present research to evaluate risk factors for vector household infestation. This study, in addition to identifying that the apartments were among the buildings with the lowest levels of infestation, showed that the presence of garden and yard (absent in the apartments) increased the risk of the presence of *Ae. aegypti* pupae [66].

The distance at which the spatial dependence was found occurring in Vila Toninho neighbourhood was higher than *Ae. aegypti* flight radius that usually does not exceed 200 m [67, 68]. This is in agreement with studies associating exposure to DENV, not only with vector-related viral dispersion, but also with human movements. Honorio et al. [29] identified that past exposure to dengue was associated with movement of people, as well as social and commercial activity. Liebman et al. [69] working with data on seroconversion and acute cases, found the cases agglomerated at distances up to 800 m. Several authors have pointed out that human movement is an important factor to be considered in the spatial dimension of virus transmission and that this movement could be more important in viral dispersion than mosquitoes [14, 29, 69, 70].

The results of this study have public health implications, both in the evaluation of the strategies currently employed and in the definition of future strategies. They are also widely applicable, since the reality found in São José do Rio Preto should not be different from the reality of many medium and large Brazilian and Latin American cities. The high seroprevalence we found also points to the ineffectiveness of the control measures being adopted and the urgent need for their re-discussion [71]. On the one hand, high seroprevalence indicates risk of cases of severe dengue and deaths; on the other, as described in Kumar and Nielsen [17] and Netto et al. [34] this points to the fact that older people could even be close to their saturation point of immunity to multiple dengue serotypes. These are different situations that also need to be considered by the control services.

Finally, the rarity of seroepidemiological studies developed in Brazil should be discussed and reviewed. The development of this type of study should be encouraged, since they produce important information and knowledge to be used in structuring appropriate surveillance systems, effective control programs and in particular to define vaccination strategies.

One of the limitations of this study is the fact that its population was not a random sample of the study area’s population but that people agreed to participate in the cohort study. This is related to the type of area chosen for its development. The fact that it was a relatively small and closed area (three of its frontiers are composed of forest, river and highway) was a matter of choice, which would facilitate the development of the work and the interpretation of the results, especially the entomological part of the study (untreated here). We also choose this area because its Basic Health Unit is linked to FAMERP, the educational institution that coordinates this research project, to serve as a foot hold for the development of the research. Even though the sample was not obtained randomly, this limitation is relieved by the fact that the households of the research participants present a distribution that covered almost all the blocks of the study area. Our sample also represented the internal distribution of households in most blocks of the study area. Only the blocks located at the southern end of the study area did not follow the spatial distribution described above.

Among the strengths of this study should be mentioned that this is one of the few carried out, despite its epidemiological importance. Another highlight is that the statistical model selected allowed the control of the spatial correlation between the individuals participating in the study so that the results obtained on the relationship between dengue seroprevalence and covariates were controlled by the geographical location of the individuals participating in the study. Moreover, the fact that the methodology we used took into account the geographical location of the participants and allowed the identification of the extent of the spatial dependence present in the study reaffirms the hypothesis that exposure to DENV is also linked with human movements.

## Conclusions

The seropositive for dengue in the study area was equal to 74.6%. Among the individuals who claimed that they never had dengue fever, 68.4% (566/828) were dengue seropositive. Older people, of a non-white ethnicity and living in a house (instead of in an apartment), adjusted for the other socioeconomic and demographic covariates considered in our study, had higher risk of a positive serological result for dengue. Spatial dependence between observations occurred until 800 m, even after adjustment for covariates, showing the necessity to consider the spatial component in the models. The use of INLA in a geostatistical approach in a Bayesian context allowed us to take into account the spatial dependence between the observations and identify the associated covariates to dengue seroprevalence.

## Additional files

Additional file 1: Combination of the separate estimates obtained from the five imputed databases for the final model (intercept, covariates and the spatial component for the imputed datasets), Vila Toninho neighbourhood, São José do Rio Preto, state of São Paulo, Brazil, 2015–2016. (DOCX 15 kb)

Additional file 2: R codes related to the analysis of the imputed data using R-INLA and a Bayesian Geostatistical approach. (R 123 kb)

Additional file 3: Deviance Information Criterion for the run models, Vila Toninho neighborhood, São José do Rio Preto, state of São Paulo, Brazil, 2015–2016. (DOCX 13 kb)

Additional file 4: Posterior means fixed effects and 95% CI, in the logit scale (betas), of the final model (intercept, covariates and the spatial component for the imputed datasets) (Imp) and the non-imputed covariate spatial model (Not imp), Vila Toninho neighborhood, São José do Rio Preto, state of São Paulo, Br, 2015–2016. (PNG 240 kb)

Additional file 5: Posterior means fixed effects and 95% CI, in the logit scale (betas), of the final model (intercept, covariates and the spatial component for the imputed datasets) (Spatial) and the imputed covariate non-spatial model (Non-spatial), Vila Toninho neighborhood, São José do Rio Preto, state of São Paulo, Brazil, 2015–2016. (PNG 229 kb)

## Abbreviations

asl: above sea level
CI: credible interval
DENV: dengue vírus
ELISA: Enzyme-linked immunosorbent assay
FAMERP: School of Medicine of São José do Rio Preto
GF: Gaussian field
GMRF: Gaussian Markov random field
INLA: Integrated Nested Laplace Appromations
OR: Odds ratio
PC: penalized priors
SIRGAS: Sistema de Referência Geocêntrico para as Américas / em inglês Geocentric Reference System for the Americas
SPDE: Stochastic partial differential equation
UTM: Universal Transverse Mercator
VIF: variance inflation factor
