# Supplementary material for: Seroprevalence for dengue virus in a hyperendemic area and associated socioeconomic and demographic factors using a cross-sectional design and a geostatistical approach, state of São Paulo, Brazil
Source: BMC Infect Dis. 2019 May 20;19:441. doi: 10.1186/s12879-019-4074-4 (PMC6528304; doi:10.1186/s12879-019-4074-4)
Supplement: Supplementary file 1 — Combination of the separate estimates obtained from the five imputed databases for the final model (intercept, covariates and the spatial component for the imputed datasets), Vila Toninho neighbourhood, São José do Rio Preto, state of São Paulo, Brazil, 2015–2016. (DOCX 15 kb) [file 12879_2019_4074_MOESM1_ESM.docx]

Additional file 1

Combination of the separate estimates obtained from the five imputed databases for the final model (intercept, covariates and the spatial component for the imputed datasets), Vila Toninho neighbourhood, São José do Rio Preto, state of São Paulo, Brazil, 2015-2016. For doing this, we used the Rubin’s rules to combine the estimates of each imputed dataset into the final ones, as follows:

- Beta: mean of betas of a specific covariate obtained for the five models (each one corresponding to one of the five imputed database obtained);
- Var: mean of the respective variances;
- B: between imputation variance;
- Var.tt: the total variance of the pooled estimated (mean variance + B);
- R: relative increase in variance due to nonresponse (B/Mean variance);
- Df: degrees of freedom for t of Student (reference) distribution;
- Se.tt: total standard error (square root of Var.tt);
- t-95%: 95% t of Student value;
- Lower and Upper 95% CI limits: 0.025 and 0.0975 quantiles considering the t Student distribution for the correspondent degrees of freedom (Df).

| Covariate | Beta | Var | B | Var.tt | R | Df | Se.tt | 95%CI | |
| --- | --- | --- | --- | --- | --- | --- | --- | --- | --- |
|  |  |  |  |  |  |  |  | Lower | Upper |
| Intercept | 0.634 | 0.089 | 0.001 | 0.089 | 0.007 | 1288 | 0.299 | 0.048 | 1.220 |
| Sex | -0.066 | 0.019 | 0.000 | 0.019 | 0.004 | 1307 | 0.138 | -0.337 | 0.204 |
| Race | 0.423 | 0.019 | 0.000 | 0.019 | 0.024 | 1099 | 0.139 | 0.150 | 0.695 |
| Marital status | -0.135 | 0.021 | 0.000 | 0.021 | 0.007 | 1292 | 0.146 | -0.421 | 0.150 |
| Age | 0.373 | 0.007 | 0.000 | 0.007 | 0.004 | 1309 | 0.085 | 0.205 | 0.540 |
| Schooling | 0.064 | 0.020 | 0.001 | 0.021 | 0.050 | 739 | 0.143 | -0.218 | 0.345 |
| Ocupation | -0.107 | 0.033 | 0.000 | 0.033 | 0.005 | 1300 | 0.183 | -0.466 | 0.251 |
| Income | -0.158 | 0.004 | 0.000 | 0.005 | 0.055 | 674 | 0.067 | -0.289 | -0.026 |
| House type | 0.524 | 0.032 | 0.000 | 0.032 | 0.003 | 1311 | 0.178 | 0.175 | 0.874 |
| Home ownership | -0.007 | 0.022 | 0.000 | 0.022 | 0.011 | 1254 | 0.149 | -0.298 | 0.285 |
| Hours at home | -0.024 | 0.022 | 0.000 | 0.022 | 0.011 | 1254 | 0.149 | -0.317 | 0.268 |
| Number of residents | 0.303 | 0.027 | 0.000 | 0.028 | 0.015 | 1209 | 0.166 | -0.024 | 0.629 |
