# Supplementary material for: Seroprevalence for dengue virus in a hyperendemic area and associated socioeconomic and demographic factors using a cross-sectional design and a geostatistical approach, state of São Paulo, Brazil
Source: BMC Infect Dis. 2019 May 20;19:441. doi: 10.1186/s12879-019-4074-4 (PMC6528304; doi:10.1186/s12879-019-4074-4)
Supplement: Supplementary file 3 — Deviance Information Criterion for the run models, Vila Toninho neighborhood, São José do Rio Preto, state of São Paulo, Brazil, 2015–2016. (DOCX 13 kb) [file 12879_2019_4074_MOESM3_ESM.docx]

Additional file 3 - Deviance Information Criterion for the run models, Vila Toninho neighborhood, São José do Rio Preto, state of São Paulo, Brazil, 2015-2016.

| Type of model | Intercept | Intercept and the covariates | | | | | |
| --- | --- | --- | --- | --- | --- | --- | --- |
|  |  | Complete database | The five Imputed databases (DB) | | | | |
|  |  |  | DB1 | DB2 | DB3 | DB4 | DB5 |
| Models without spatial component | | | | | | | |
|  | 1500.8 | 1058.9 | 1463.9 | 1466.7 | 1466.8 | 1464.0 | 1466.3 |
| Models with spatial component and without interactions | | | | | | | |
|  | 1456.1 | 1039.7 | 1437.2 | 1439.1 | 1438.7 | 1437.2 | 1439.7 |
| Models with spatial component and with interactions (race, age and house type) | | | | | | | |
| Considered interactions | Three and all two interactions | | 1443.5 | 1445.4 | 1445.5 | 1443.4 | 1446.0 |
|  | All two interactions | | 1441.9 | 1443.9 | 1443.6 | 1442.0 | 1444.5 |
|  | Race x age and race x house type | | 1441.0 | 1443.0 | 1442.6 | 1441.0 | 1443.5 |
|  | Race x age and age x house type | | 1439.9 | 1442.0 | 1441.7 | 1440.1 | 1442.6 |
|  | Race x house type and age x house type | | 1440.2 | 1442.0 | 1441.7 | 1440.1 | 1442.7 |
|  | Race x age | | 1439.1 | 1441.1 | 1440.7 | 1439.2 | 1441.6 |
|  | Race x house type | | 1439.2 | 1441.0 | 1440.7 | 1439.1 | 1441.6 |
|  | Age x house type | | 1438.2 | 1440.1 | 1439.8 | 1438.2 | 1440.7 |
