# Supplementary figures and images for: Seroprevalence for dengue virus in a hyperendemic area and associated socioeconomic and demographic factors using a cross-sectional design and a geostatistical approach, state of São Paulo, Brazil
Source: BMC Infect Dis. 2019 May 20;19:441. doi: 10.1186/s12879-019-4074-4 (PMC6528304; doi:10.1186/s12879-019-4074-4)

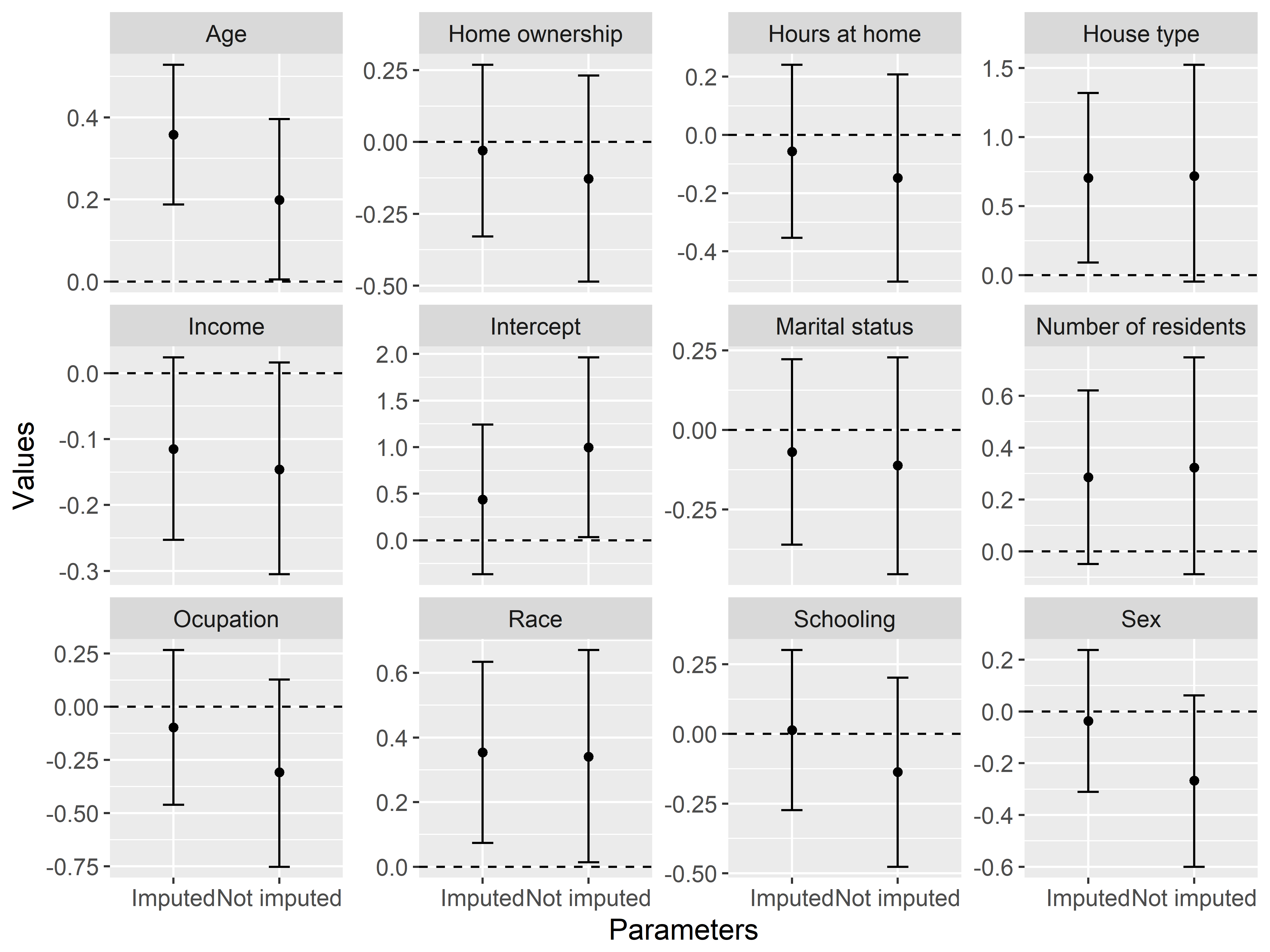

Supplement: Supplementary file 4 — Posterior means fixed effects and 95% CI, in the logit scale (betas), of the final model (intercept, covariates and the spatial component for the imputed datasets) (Imp) and the non-imputed covariate spatial model (Not imp), Vila Toninho neighborhood, São José do Rio Preto, state of São Paulo, Br, 2015–2016. (PNG 240 kb) [file 12879_2019_4074_MOESM4_ESM.png]

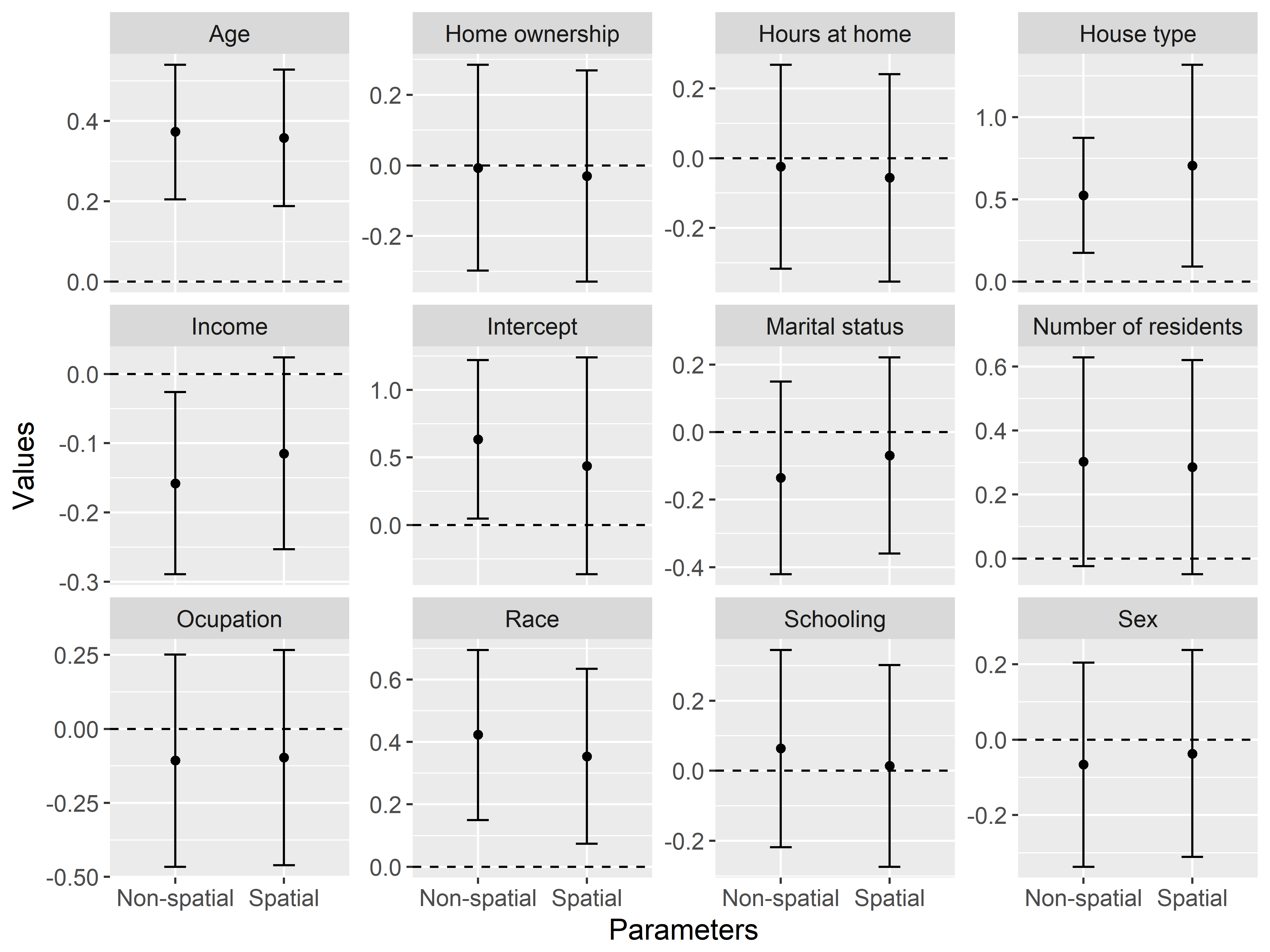

Supplement: Supplementary file 5 — Posterior means fixed effects and 95% CI, in the logit scale (betas), of the final model (intercept, covariates and the spatial component for the imputed datasets) (Spatial) and the imputed covariate non-spatial model (Non-spatial), Vila Toninho neighborhood, São José do Rio Preto, state of São Paulo, Brazil, 2015–2016. (PNG 229 kb) [file 12879_2019_4074_MOESM5_ESM.png]
